# IGF1 and NRG1 Enhance Proliferation, Metabolic Maturity, and the Force-Frequency Response in hESC-Derived Engineered Cardiac Tissues

**DOI:** 10.1155/2017/7648409

**Published:** 2017-08-29

**Authors:** Cassady E. Rupert, Kareen L. K. Coulombe

**Affiliations:** Center for Biomedical Engineering, School of Engineering, Brown University, Providence, RI 02912, USA

## Abstract

Insulin-like growth factor 1 (IGF1) and neuregulin-1*β* (NRG1) play important roles during cardiac development both individually and synergistically. In this study, we analyze how 3D cardiac tissue engineered from human embryonic stem cell- (hESC-) derived cardiomyocytes and 2D-plated hESC-cardiomyocytes respond to developmentally relevant growth factors both to stimulate maturity and to characterize the therapeutic potential of IGF1 and NRG1. When administered to engineered cardiac tissues, a significant decrease in active force production of ~65% was measured in all treatment groups, likely due to changes in cellular physiology. Developmentally related processes were identified in engineered tissues as IGF1 increased hESC-cardiomyocyte proliferation 3-fold over untreated controls and NRG1 stimulated oxidative phosphorylation and promoted a positive force-frequency relationship in tissues up to 3 Hz. hESC-cardiomyocyte area increased significantly with NRG1 and IGF1 + NRG1 treatment in 2D culture and gene expression data suggested increased cardiac contractile components in engineered tissues, indicating the need for functional analysis in a 3D platform to accurately characterize engineered cardiac tissue response to biochemical stimulation. This study demonstrates the therapeutic potential of IGF1 for boosting proliferation and NRG1 for promoting metabolic and contractile maturation in engineered human cardiac tissue.

## 1. Introduction

The development of defined methods to derive cardiomyocytes from human pluripotent stem cells (hPSCs) has provided a valuable platform to develop regenerative medicine technologies. Cardiomyocytes derived from human embryonic stem cells (hESC-cardiomyocytes) and tissues constructed from them have been shown to exhibit a cardiac phenotype characterized by gene expression patterns, electrophysiological behavior, and mechanical function. Previous research describes the response of engineered cardiac tissues (ECTs) to common drugs; however, little is known about how ECTs develop under in utero-like biochemical conditions. Just as embryonic development has informed directed differentiation of hESCs to the cardiac lineage, we hypothesize that fetal development can inform how ECTs grow and mature. In order for ECTs to advance towards the clinical realm as both a translational therapy and an *in vitro* model, a more thorough understanding of how to manipulate hESC-cardiomyocyte maturation in 3D tissues via developmental cues is required.

Two growth factors crucial to cardiac development *in vivo* are insulin-like growth factor 1 (IGF1) and neuregulin-1*β* (NRG1). IGF1 has been implicated in physiological growth of the heart, and studies performed to downregulate [1] and upregulate [2] its receptor in mouse models show dilated cardiomyopathy and cardiomyocyte physiological hypertrophy, respectively. At the cellular level, IGF1 has been shown to increase proliferation in hESC-cardiomyocytes via the PI 3-kinase/Akt pathway *in vitro* [3]. Thus, carefully regulated IGF1 signaling is required for proper development and maturation of the heart in order to achieve both cardiomyocyte proliferation and growth, yet the timing of IGF1 stimulation likely alters this response. Similarly, a critical need for appropriate NRG1 signaling during development has been demonstrated. When NRG1 [4] or its receptors ErbB2 [5] and ErbB4 [6] are deleted in mice, ventricular trabeculation and endocardial cushion formation are depressed, and mice die midembryogenesis. When mice embryos are cultured ex vivo, NRG1 is required for complete cardiac conduction system development [7]. A synergistic effect of IGF1 and NRG1 has also been reported in mice in utero where their combined presence is necessary for ventricular wall expansion and atrioventricular cushion formation [8]. We hypothesized that we could improve functional maturation of engineered cardiac tissue formed from hESC-cardiomyocytes by extending the developmental period beyond the stereotypical two-week differentiation time point via application of developmental growth factors IGF1 and NRG1 independently or in combination after formation of 3D hESC-derived engineered cardiac tissues.

In the current study, we demonstrate that our ECTs are sensitive to biochemical stimulation with IGF1 and NRG1 and that hESC-cardiomyocytes in 3D tissues respond in unique patterns to these growth factors, which is not predicted from previous studies. We show that force production declines by 60–70% in ECTs with IGF1 and/or NRG1 stimulation. However, we discovered that our ECTs respond sensitively to IGF1 and NRG1 stimulation in proliferative activity and metabolic capacity, respectively, and that the force-frequency relationship is preserved or improved with NRG1 or NRG1 + IGF1, respectively. We show that hESC-cardiomyocytes exhibit increased area when plated in 2D and treated with NRG1 or IGF1 + NRG1 and that this 2D “hypertrophy” does not correlate with increased force in 3D tissues. These data suggest that 2D and transgenic mouse models are not sufficient to fully predict the effects of biochemical stimulation on 3D hESC-derived engineered cardiac tissues and that our platform for forming and characterizing ECTs uniquely possesses the sensitivity to describe tissue response to physiologically relevant growth factors for regenerative medicine applications.

## 2. Materials and Methods

### 2.1. Cell Culture and Differentiation

**Figure 1:**
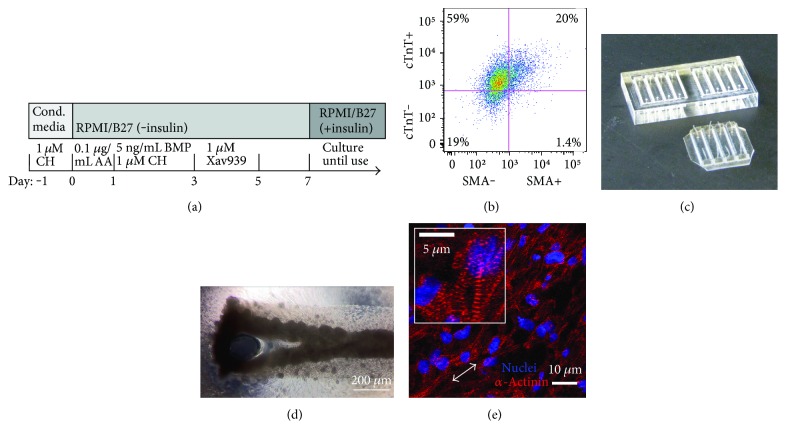
Directed differentiation of hESCs result in highly enriched cardiomyocyte populations which form beating tissues *in vitro*. (a) Timeline of differentiation of cardiomyocytes from hESCs (Cond. media: MEF-conditioned media, CH: Chiron99021, AA: activin A, BMP: bone morphogenic protein 4). (b) Differentiated populations were immunofluorescently stained for cardiac troponin T (cTnT) and smooth muscle actin (SMA) and analyzed with flow cytometry. Cardiomyocytes cultured with a collagen-1 matrix in PDMS molds cast from custom laser-etched acrylic negatives (c) remodeled the matrix and formed beating constructs within 48 hours (d). (e) Immunofluorescence of *α*-actinin (red) and nuclei (blue) in engineered tissue sections. Myofibril striations were visible (inset). White double-headed arrow indicates direction of uniaxial stress in tissue during culture.

**Figure 2:**
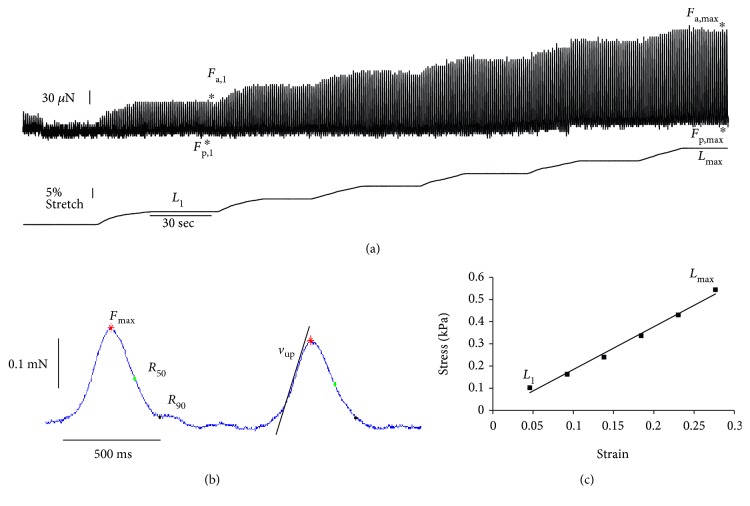
Engineered muscle displays robust twitch contractions. (a) Force (top) and length (bottom) trace of an ECT stretched to 130% of its initial length in 5% steps with individual twitch contractions recorded at each length. Active and passive force are indicated at 5% stretch (length step *L*_1_) as *F*_a,1_ and *F*_p,1_ and at 30% stretch (*L*_max_) as *F*_a,max_ and *F*_p,max_ as indicated by asterisks. (b) Traces of individual contractions were processed to identify maximal twitch stress (*σ*_a,max_, red asterisk), time to 50% and 90% force relaxation (*R*_50_, green asterisk, and *R*_90_, black asterisk), and peak upstroke velocity (*v*_up_). (c) Young's modulus (stiffness) of each tissue was determined by measuring passive stress, *σ*_p_, from 5% stretch of initial length (*L*_1_ = 0.05 strain) to 30% stretch (*L*_max_ = 0.30 strain) and calculating the slope of the line of best fit. In this example, Young's modulus = 1.93 kPa (*R*^2^ = 0.99).

**Figure 3:**
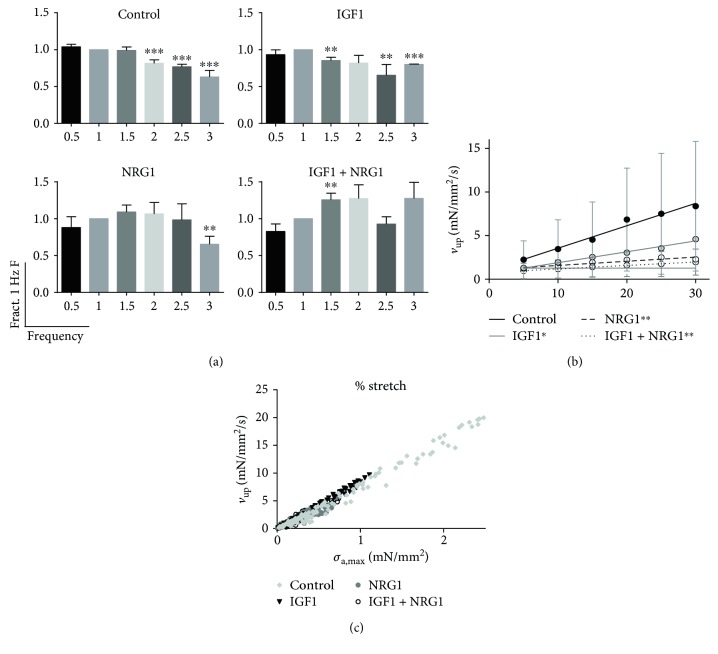
Engineered muscle displays a force-frequency response that is sensitive to biochemical stimulation. (a) Maximum active stress at 30% stretch was measured at increasing frequencies for control, IGF1, NRG1, and IGF1 + NRG1. Data are shown normalized to active stress at 1 Hz and expressed as mean ± SEM. (b) The relationship between upstroke velocity (*v*_up_) and percent stretch was plotted and fit with a regression line for each treatment group. Data are expressed as mean ± SEM at each length. (c) A scatterplot of *v*_up_ versus maximum active stress, *σ*_a,max_, was plotted. *n* = 10 per group. ^∗^*P* < 0.05, ^∗∗^*P* < 0.01, and ^∗∗∗^*P* < 0.001 versus 1 Hz of the same group (a) or slope of the line versus control (b).

**Figure 4:**
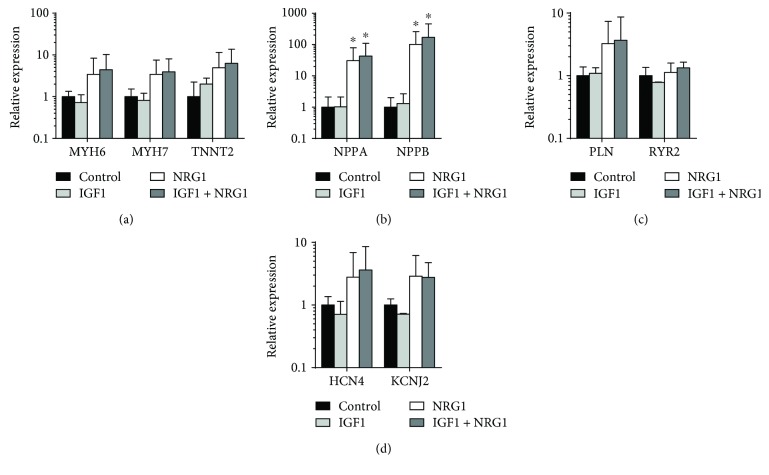
Engineered tissues alter their gene expression patterns by q-RT-PCR analysis in response to biochemical stimulation. Genes shown encode proteins associated with the contractile lattice (MYH6: *α*-myosin heavy chain, MYH7: *β*-myosin heavy chain, TNNT2: cardiac troponin T) (a), development (NPPA: atrial natriuretic peptide, BNNP: brain natriuretic peptide) (b), calcium handling (PLN: phospholamban, RYR2: ryanodine receptor 2) (c), and voltage-gated ion channels (HCN4: hyperpolarization-activated cyclic nucleotide-gated channel, KCNJ2: inward-rectifier potassium ion channel) (d). *n* = 3 per group; data are shown as fold induction of gene expression normalized to HPRT1 and expressed as mean ± SEM. ^∗^*P* < 0.05.

**Figure 5:**
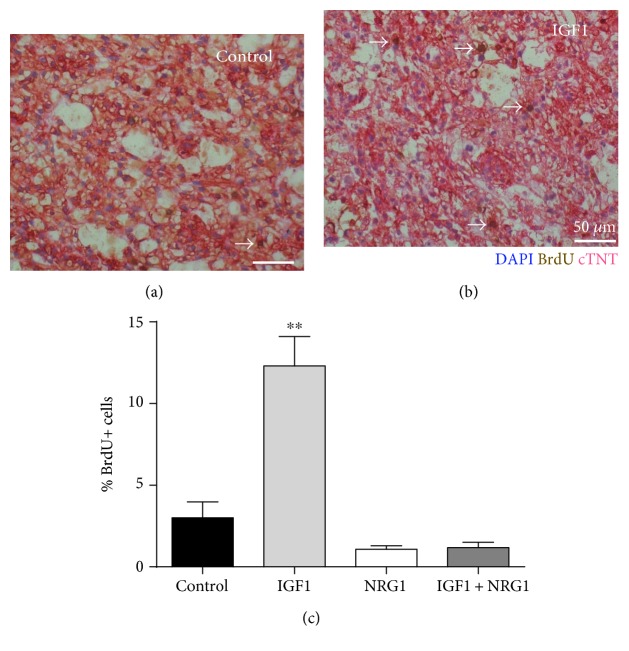
IGF1 stimulates cardiomyocyte proliferation in engineered tissues. Immunohistological staining of cardiac troponin T (pink), nuclei (blue), and bromodeoxyuridine- (BrdU-) positive nuclei (brown) in control (a) and IGF1-treated (b) ECTs. (c) Percent BrdU-positive nuclei was calculated for each group by counting the number of BrdU-positive cardiac nuclei of the total cardiac nuclei (*n* = 3 biological replicates with ≥100 cardiomyocytes counted per group). ^∗∗^*P* < 0.01.

**Figure 6:**
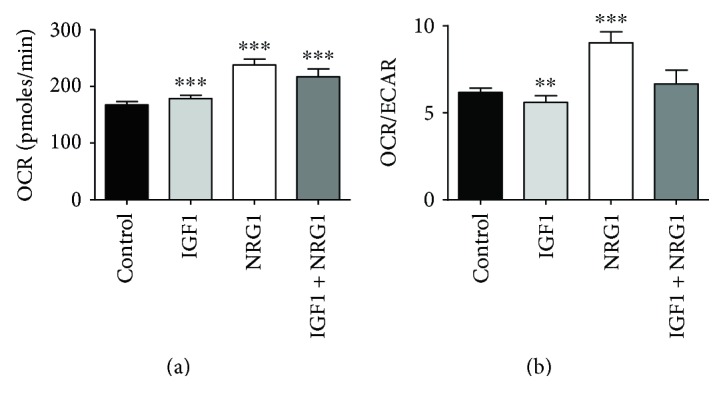
NRG1 treatment increases mitochondrial activity in cardiomyocytes. Basal metabolic profiling of hESC-cardiomyocytes was normalized to live cells (with LIVE/DEAD® staining) to determine oxygen consumption rate (OCR) (a), extracellular acidification rate (ECAR), and the OCR/ECAR ratio (b) (*n* = 6). Data are expressed as mean ± SD. ^∗∗^*P* < 0.01 and ^∗∗∗^*P* < 0.001 versus control.

**Figure 7:**
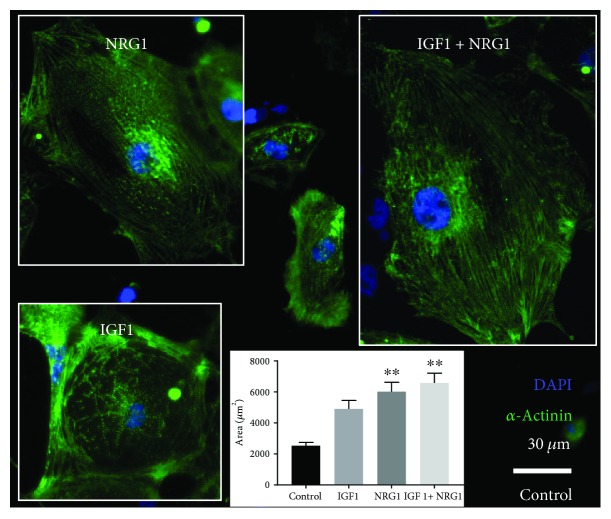
NRG1 treatment stimulates growth in single hESC-cardiomyocytes. Immunofluorescence of *α*-actinin (green) and nuclei (blue) in control (center), IGF1 (bottom left), NRG1 (top left), or IGF1 + NRG1 (top right) treated hESC-cardiomyocytes. Area was measured for each group (bottom center). *n* ≥ 38 cells analyzed per group; data are expressed as mean ± SEM. ^∗∗^*P* < 0.01.

Undifferentiated RUES2 human embryonic stem cells (hESCs) from Rockefeller University were maintained in mouse embryonic-fibroblast conditioned media, supplemented with basic fibroblast growth factor (R&D Systems). HESCs were differentiated into cardiomyocytes with a previously described directed differentiation protocol (Figure 1(a) [9]). Briefly, CHIR99021 (Cayman Chemical), activin A, bone morphogenetic protein 4 (BMP4; R&D Systems), and tankyrase inhibitor XAV 939 (Tocris Bioscience) were applied sequentially in defined, monolayer culture conditions on Matrigel™- (BD Biosciences) coated 6-well plates [10]. HESC-cardiomyocytes were differentiated in RPMI 1640 + 1X B27 supplement without insulin (RPMI/B27 without insulin; Life Technologies) and maintained in RPMI/B27 (with insulin) with medium replaced every two days. After two weeks of differentiation, hESC-cardiomyocytes were harvested with 0.25% trypsin in 0.5 M EDTA (Life Technologies) and either incorporated into engineered tissues (Figures 1–5) or cryopreserved [11] for later use in single-cell experiments (Figures 6 and 7).

### 2.2. Flow Cytometry

Cardiac purity was determined for each hESC-cardiomyocyte harvest. Cells were fixed in 4% PF and stained with antibodies against cardiac troponin T (cTnT, 2 *μ*g/mL; Thermo Fisher Scientific) and alpha-smooth muscle actin (*α*-SMA, 0.5 *μ*g/mL; Abcam) to identify cardiomyocytes (cTnT+), fibroblast-like cells (SMA+), and immature cardiomyocytes (double positive for cTnT and SMA [12]). Samples were analyzed with a FACSAria II cell sorter (BD Biosciences) and 10,000 events were recorded per sample. FACS data was analyzed used FlowJo software (Figure 1(b)) where gates were set based on isotype antibody controls.

### 2.3. Mold and Tissue Formation

Custom acrylic and polydimethylsiloxane (PDMS) molds were produced for ECT culture as previously reported [13]. Negative templates for tissue molds were created using a 100 W CO_2_ laser to etch a pattern generated in Adobe Illustrator into 1/4” acrylic (TAP Plastics). Polydimethylsiloxane (PDMS; Thermo Fisher Scientific) was poured into acrylic negatives, degassed, and cured overnight at 60°C (Figure 1(c)). Tissue molds were sterilized by autoclave and made hydrophilic with a BD-20A high-frequency generator (Electro-Technic Products). Engineered tissues were created by combining 1 × 10^6^ hESC-cardiomyocytes 50%/50% vol/vol with 2.5 mg/mL rat tail collagen-I (Advanced BioMatrix) for a final concentration of 1.25 mg/mL collagen-I and pipetting into the prepared molds. Collagen-cell suspensions were allowed to gel for 45 minutes at 37°C before RPMI/B27 was added. Tissues were cultured in 6-well plates fitted with C-pace stimulator lids (IonOptix) and stimulated at 1 Hz, 4 ms pulse width, 5 V/cm for 14 days immediately following tissue formation. Tissues contracted the collagen and began beating within 48 hours of formation (Figure 1(d)). Experimental groups were treated daily with 100 nM [3] insulin-like growth factor (PeproTech), 100 ng/mL [14] neuregulin-1 (R&D Systems), or both 100 nM IGF1 and 100 ng/mL NRG1. All experiments described used these concentrations where relevant. Media was replaced every other day, and tissues were cultured for two weeks. All constructs were treated with 10 *μ*M bromodeoxyuridine (BrdU; Roche) 18–24 hours prior to fixation to label replicating DNA.

### 2.4. Mechanical Testing

Cardiac tissues were cut into strips and their passive and active mechanical properties were measured with a custom mechanics setup as previously described [15, 16]. Tissue strips were mounted on two hooks (attached to a force transducer and motor arm), bathed in Tyrode's solution containing 1.8 mM Ca^2+^ at 30–34°C, and electrically field stimulated via platinum electrodes. Force exerted by the tissue was measured with a 100 mN load cell (Aurora Scientific). Tissues were initially stretched to *L*_0_, determined as the shortest length at which individual contractions could be detected by the force transducer. The tissues were then stretched by steps of 5% *L*_0_ to 30% stretch (Figure 2(a)). Once at 30% stretch, tissues were paced at increasing frequencies and the fastest frequency they were able to follow was measured and recorded as the maximum capture rate (MCR). Mechanical experiments were run in biological triplicate with three to four replicates in each group.

The following calculations were made based on the data obtained from mechanical testing. Active stress, *σ*_a_, was determined at each length by averaging the amplitude of twitch force, *F*_a_, of at least 5 contractions and normalizing by the cross-sectional area (CSA). The CSA of each tissue strip was assumed to be an ellipse with the measured preparation width and height corresponding to the major and minor axes, respectively. The fold increase in active stress was calculated from the active stress (*σ*_a,1_) at the first length step (*L*_1_ = 1.05 *L*_0_) and maximum active stress (*σ*_a,max_) at 30% stretch (*L*_6_ = *L*_max_ = 1.30 *L*_0_) using the following equation (Figure 2(a)):

(1) $$\begin{array}{c} Fold\;increase=\frac{\sigma_{a,\text{max}}-\sigma_{a,1}}{\sigma_{a,1}}. \end{array}$$

Stiffness of each tissue strip was calculated by plotting the passive stress produced by each tissue against the strain (Figure 2(c)). Passive stress, *σ*_p_, was calculated by normalizing the passive (or baseline) force produced by the tissue at each step, *F*_p_, by the CSA and is reported in units of kPa. Strain, *ε*_x_, at each step, *L*_x_, was determined by the following equation:

(2) $$\begin{array}{c} \varepsilon_{x}=\frac{L_{x}-L_{0}}{L_{0}}. \end{array}$$

A linear relationship of these data allowed for a regression line to be fit to each stress versus strain plot (*R*^2^ values of 0.749 to 0.998), and the stiffness (Young's modulus) is reported as the slope of the line for each ECT strip. Passive stiffness was difficult to measure in tissues due to uncontrolled drift in the baseline force during testing of some samples, resulting in reduced sample sizes of *n* = 5 − 6.

Contraction kinetics were calculated from data traces acquired at 1000 Hz sampling rate in custom LabVIEW software using a custom analysis script in MATLAB®. The peak speed of force development during contraction, upstroke velocity (*v*_up_), was determined as the slope of the linear regression line to the steepest rise in stress and was averaged across at least 5 contractions (Figure 2(b)). The peak force of each contraction and the time to relax to 50% and 90% (*σ*_a,max_, *R*_50_, and *R*_90_, resp.) were calculated and averaged across at least 5 contractions (Figure 2(b)).

### 2.5. Immunofluorescent and Immunohistochemical Staining

Engineered tissues were fixed in 4% paraformaldehyde (PF; Sigma-Aldrich), embedded in paraffin, and sectioned at 5 *μ*m. Sections were blocked in 1.5% normal goat serum in PBS, and primary antibodies for cardiac troponin T (cTnT) to label cardiomyocytes (0.5 *μ*g/mL; Thermo Fisher Scientific) and BrdU for cells in the S phase of the cell cycle (1.5 U/mL; Roche) were applied sequentially overnight with development using Vector Red and DAB (Vector Laboratories) according to manufacturer's instructions to color cTnT-positive cells pink and BrdU-positive nuclei brown. Hematoxylin (7 mg/mL; Sigma-Aldrich) was used as a nuclear counterstain. For cardiomyocyte proliferation studies, at least 100 cTnT-positive nuclei were counted per slide with proliferating cardiomyocytes identified as those with brown nuclei and senescent cardiomyocytes those with blue nuclei. Brightfield images were taken with an Olympus IX70 Inverted Microscope.

For immunofluorescent staining of ECT sections, slides first underwent antigen retrieval with a Proteinase K digest (10 *μ*g/mL; Roche) for 12 minutes at 37°C. Mouse anti-alpha-actinin was used to identify cardiomyocyte z-discs of the myofibrils (1 : 800; Sigma-Aldrich) with Alexa Fluor® 488 goat anti-mouse secondary antibody (Life Technologies) and Hoechst 33342 to label nuclei (1.5 *μ*g/mL; Sigma-Aldrich; Figure 1(d)). The same protocol was used for immunofluorescent staining of single cells with the Proteinase K digest replaced by a permeablization step in Triton X-100 (0.25%; Sigma-Aldrich) for 6 minutes at room temperature. Confocal images were taken with a Zeiss LSM 510 Meta Confocal Laser Scanning Microscope. Image processing and analysis was performed with ImageJ [17], CellProfiler [18], and MATLAB (MathWorks).

### 2.6. RNA Isolation and Quantitative Reverse Transcriptase-Polymerase Chain Reaction

mRNA was extracted from engineered tissues using the RNeasy Mini Kit (QIAGEN), and mRNA concentration was measured with a NanoDrop 1000 Spectrophotometer (Thermo Fisher Scientific). cDNA was synthesized from the same mass of mRNA per sample with the SuperScript III First-Strand Synthesis System (Life Technologies) and included genomic DNA digestion. cDNA samples were combined with primers (Thermo Fisher Scientific) and SYBR Master Mix (Life Technologies), and quantitative real-time PCR was run with an Applied Biosystems® 7900 fast real-time system (Life Technologies). Relative expression levels were calculated using the 2^(−ΔΔCt) [19] with HPRT as an internal control (Figure 4). Samples were run with biological and technical triplicates.

### 2.7. Mitochondrial Content Quantification

Single hESC-cardiomyocytes were plated on Matrigel-coated chamber slides (Thermo Fisher Scientific) at 22 × 10^3^ cells/cm^2^ on day 0, and treatment groups were pulsed daily with IGF1, NRG1, or IGF1 + NRG1. On day 5, cells were treated with MitoTracker® Red FM (Life Technologies), incubated for 30 minutes at 37°C, and then fixed in 4% PF. Cells were imaged with an Eclipse Ti-E inverted fluorescence microscope (Nikon), and total integrated fluorescence per image, normalized by cell number, was calculated with a custom CellProfiler Pipeline. CellProfiler data was analyzed in MATLAB.

### 2.8. Metabolic Activity

HESC-cardiomyocytes were seeded at 40 × 10^3^ cells/well of a Matrigel-coated XFe96 cell culture microplate (Seahorse Bioscience, North Billerica, MA, USA) and fed daily for two weeks. Treatment groups were supplemented with IGF1, NRG1, or NRG1 + IGF1 daily. On day 14, cells were switched to 175 *μ*L/well assay media: XF Base Medium (Seahorse Bioscience), 4 mM GlutaMAX, 1 mM sodium pyruvate (Life Technologies), and 25 mM glucose (Sigma-Aldrich) and incubated for 90 minutes at 0% CO_2_ and 37°C. Basal oxygen consumption rate (OCR) and extracellular acidification rate (ECAR) measurements were then made with an XFe96 Extracellular Flux Analyzer (Seahorse Bioscience).

### 2.9. Statistical Analysis

Data are expressed as mean ± SEM or SD as indicated in figure legends. Statistical significance was determined by using the Student unpaired *t*-tests (Figures 3(a) and 3(b)), one-way ANOVA with Dunnett's multiple comparisons (Figures 4–7), linear regression analysis (Figure 3(b)), or as indicated in the text. A *P* value < 0.05 was considered statistically significant.

## 3. Results

### 3.1. Cardiac Force-Length Relationship Is Recapitulated in ECTs

ECTs were formed in a collagen gel with 1.0 × 10^6^ cells per tissue ranging from 81 to 95% cardiac purity by flow cytometry (Figure 1(b)) in custom PDMS molds (Figure 1(c)). We treated ECTs daily with 100 nM IGF1, 100 ng/mL NRG1, or both 100 nM IGF1 + 100 ng/mL NRG1 (IGF + NRG) and continuously paced ECTs at 1 Hz during their two-week culture period. ECTs contracted within the PDMS molds (Figure 1(d)) and assembled striated myofibrils (Figure 1(e)). To characterize the effects of biochemical treatment with IGF1 and NRG1 upon contractile function of ECTs, we used a custom mechanics apparatus to measure intact twitch forces and their kinetics under electrical stimulation. ECTs from all groups followed electrical stimulation at 1 Hz both in culture and during mechanical measurements; however, ECTs treated with NRG1 or IGF1 + NRG1 exhibited a loss of spontaneous contractions (automaticity) when external pacing was paused after just one week in ECT culture. ECTs exhibited the classic Frank-Starling relationship [20] with active twitch force, *F*_a_, increasing as tissues were stretched up to 30% of their initial length, *L*_0_ (Figure 2(a)). A fold change in active stress significantly greater than 1 indicates a positive force-length relationship. This signifies increased contractile force with stretch, consistent with the Frank-Starling relationship (Table 1). There was no significant difference in fold-change of active stress between control and treatment groups, indicating that biochemical stimulation neither enhanced nor inhibited the sensitivity of hESC-cardiomyocytes to increases in length. Passive tissue stiffness, defined as passive force normalized by cross-sectional area, ranged from 0.685 ± 0.265 kPa in control to 0.964 ± 0.158 kPa in NRG1-treated tissues (Figure 2(c), Table 1). The maximum passive stiffness value measured, 1.928 kPa, was equal to that previously reported for scaffold-free engineered hESC-cardiac patches [16] and about tenfold below that of human myocardium (~16 kPa) [21].

### 3.2. Active Contractile Force and Force-Frequency Relationship Are Sensitive to Biochemical Stimulation

We hypothesized that cardiomyocyte maturation due to biochemical stimulation would be detected at the level of force production in ECTs. To test this, we measured active stress in tissue preparations up to 30% stretch, *σ*_a,max_ (Table 1). Surprisingly, active stress was significantly decreased by 63%, 64%, and 65% of control with IGF1, NRG1, and IGF1 + NRG1 treatment, respectively. The average *σ*_a,max_ in the control group, 0.59 ± 0.14 mN/mm^2^, is equivalent to other reported values in collagen-based engineered hESC-cardiac tissue [22] and two orders of magnitude below healthy human myocardium [23]. The force-frequency relationship, which is a positive relationship *in vivo* and one often missing *in vitro* [22, 24, 25], was examined in our ECTs by calculating the fraction of the force produced at 1 Hz to that produced when pacing tissues at a range of stimulation frequencies (Figure 3(a)). We observed a negative force-frequency response in control and IGF1-treated tissues with significant decreases in force beyond 1.5 Hz and 1 Hz, respectively. In NRG1-treated tissues, we observed sustained force amplitude with increasing frequency and measured a significant drop in force only at 3 Hz (180 beats per minute), which is close to the maximum rate captured. IGF1 + NRG1-treated tissues showed the most positive force-frequency relationship, with force significantly higher at 1.5 Hz and no significant reduction in force with increasing frequency, similar to recently reported results when using electromechanical stimulation [26, 27]. Lastly, the maximum capture rate (MCR) of each group was determined at 30% stretch (Table 1). The IGF1-treated group had the slowest MCR, while the NRG1-treated group had the fastest MCR at 2.8 ± 0.3 Hz which was significantly greater than that of the control.

### 3.3. Biochemical Stimulation Does Not Alter the Kinetics of Contraction and Relaxation

To determine if biochemical stimulation altered contraction and relaxation kinetics independent of changes in force, we examined the speed of tissue contraction and relaxation. Peak rate of force development or upstroke velocity, *v*_up_ (Figure 2(c)), at 30% stretch was fastest in the control group, depressed with IGF1 treatment, and significantly slower in the NRG1 and IGF1 + NRG1-treated groups (Table 2). The change in *v*_up_ with stretch was then examined, and a slope significantly greater than 0 was observed in all groups, indicating a significant increase in *v*_up_ with stretch as is expected for increased twitch force amplitude. The slope was steepest in the control group and significantly reduced in treatment groups (Figure 3(b)). The control group had the slowest time to 50% and 90% relaxation from peak force (*R*_50_ and *R*_90_, resp.; Figure 2(b)) and both *R*_50_ and *R*_90_ were decreased with NRG1 treatment (Table 2). However, the apparent changes in contraction upstroke velocity and relaxation kinetics with biochemical stimulation at maximal stretch can be explained by the lower maximal twitch force that biochemically stimulated tissues produced. Because force production in treated ECTs was 35% of control, we also examined *v*_up_*, R*_50_, and *R*_90_ in control ECTs at 35% of their maximal force production (at 10% stretch) where *R*_50_ and *R*_90_ were very similar to what has been previously reported for hESC-cardiomyocytes [28]. No significant differences were observed between treatment groups and 35% control (Table 2). When examined over the range of maximal stress produced across all groups (Figure 3(c)), *v*_up_ does not differ with IGF1 and NRG1 treatment. This confirms that for a given *σ*_a,max_, contraction kinetics are not altered with biochemical stimulation.

### 3.4. Biochemical Stimulation Induces Increased Gene Expression of Maturation Markers in ECTs

To determine if our biochemical stimulation could induce changes at the molecular level in our ECTs, we used q-RT-PCR to examine mRNA expression levels of a panel of cardiac markers involved in force production, development, calcium handling, and resting membrane potential. Genes MYH6, MYH7, and TNNT2, which code for proteins *α*- and *β*-myosin heavy chain and cardiac troponin T, respectively, that are components of the contractile lattice increased expression by about 4- to 6-fold without reaching significance when NRG1 was included in the stimulation cocktail (Figure 4(a)). Atrial and ventricular myosin light chain expression did not change significantly with stimulation (data not shown). NPPA and NPPB, coding for atrial (ANP) and brain (BNP) natriuretic peptides, had expression levels greater than 50-fold above control with NRG1 stimulation (Figure 4(b)). Their increased expression has been associated with cardiovascular development, where natriuretic peptides are expressed in the ventricles during gestation and in the cardiac conduction system after birth [29]. Phospholamban (PLN), whose low expression is characteristic of stem cell-derived cardiomyocytes but is required for proper calcium handling [30], also trended towards increased expression with NRG1 and IGF1 + NRG1 treatment, which may contribute to decreased twitch amplitude in those groups. Ryanodine receptor 2 (RYR2), responsible for the release of Ca^2+^ from the sarcoplasmic reticulum, was not altered by treatment (Figure 4(c)). Two ion channels important for resting membrane potentials in cardiomyocytes were not altered by biochemical stimulation, namely, the hyperpolarization-activated channel 4 (HCN4), responsible for the “funny current” and necessary for pacemaker action potentials in cardiomyocytes [31], and the KCNJ2 gene, encoding the inward-rectifier potassium ion channel, K_ir_2.1 (Figure 4(d)). No gene expression differences were observed with IGF1 treatment alone, and synergy between NRG1 and IGF1 was not observed. Together, this gene expression data suggests that the cardiac lineage may be pushed forward into a more mature phenotype in ECTs stimulated with NRG1.

### 3.5. IGF1 Increases Cardiomyocyte Proliferation in ECTs

Because force production was decreased in biochemically stimulated ECTs, we sought to identify alternative molecular mechanisms occurring in IGF1- and NRG1-treated tissues. First, we examined proliferation rates in ECTs by quantifying the number of cardiomyocytes that had incorporated bromodeoxyuridine (BrdU) into their nuclei during DNA replication (S phase of the cell cycle; Figures 5(a) and 5(b)). This was done by incubating ECTs with BrdU overnight, prior to fixation, and staining tissue sections for cardiac troponin T and BrdU. Proliferation rates increased more than 3-fold over control with IGF1 treatment from 3.0 ± 0.6% to 12.3 ± 1.0% (Figure 5(c)). NRG1 treatment negated this effect, returning proliferation rates to that of control. As seen in both gene expression and mechanics data, NRG1 dominates the treatment effects when administered simultaneously with IGF1. Because treatment of hESC-cardiomyocytes with IGF1 in 2D culture results in increased cytokinesis, not karyogenesis, we hypothesize the same is the case in ECTs.

### 3.6. Cellular Metabolic Activity Is Modulated by NRG1 and IGF1 Treatment

Because NRG1 signaling has been implicated in altering cellular respiration [32] and because we noticed increased media yellowing of ECTs treated with NRG1 and IGF1 + NRG1, we investigated mitochondrial content and metabolic rates in hESC-cardiomyocytes. We first sought to confirm that overall mitochondria content was differentially affected by growth factor stimulation. Plated single cells were treated for 2 or 5 days with IGF1, NRG1, or IGF1 + NRG1, and mitochondrial content per cell was then determined with MitoTracker Red. No differences were seen between groups treated for 2 days, but 5 days of NRG1 treatment significantly increased mitochondrial content 3.6 ± 0.6-fold over control (*P* < 0.05, *n* = 4). Both increased mitochondria size or biogenesis could explain this result, so we next sought to assess if increased mitochondrial content in NRG1-treated cells was associated with an increase in metabolic activity; we examined basal cardiomyocyte metabolic rates with an XFe96 Extracellular Flux Analyzer (Seahorse Bioscience). To emulate the 3D engineered tissue conditions as closely as possible, we plated cardiomyocytes as a confluent monolayer and treated for 2 weeks with IGF1, NRG1, or IGF1 + NRG1 daily. On day 14, we measured the basal oxygen consumption rate (OCR, Figure 6(a)), extracellular acidification rate (ECAR), and calculated the ratio of the two measures (OCR/ECAR, Figure 6(b)) with all measures normalized to cell number. A significant increase in OCR over control was present in all treatment groups, indicating increased metabolic activity with each treatment. The OCR/ECAR ratio indicates relative levels of mitochondrial respiration and glycolysis, with an increased ratio reflecting an increase in mitochondrial respiration and a decreased ratio reflecting an increase in glycolysis. The IGF1-treated cells had a significantly lower OCR/ECAR ratio versus control, whereas NRG1-treated cells had a significantly higher OCR/ECAR ratio. IGF1 + NRG1-treated cells displayed a trend towards an increased OCR/ECAR ratio, but this did not reach significance. These results align with previous studies showing that proliferating cells have increased rates of glycolysis [33] and treatment with NRG1 increases oxidative phosphorylation subunits in mitochondria of rat skeletal muscle cells [34].

### 3.7. NRG1 Induces Increased Area at the Single-Cardiomyocyte Level

Because both IGF1 and NRG1 have been implicated in cardiomyocyte hypertrophy through traditional 2D culture methods using single cells [35, 36], we examined the morphological response of single hESC-cardiomyocytes plated in 2D to IGF1 and NRG1. Cells were treated daily with IGF1, NRG1, IGF1 + NRG1, or a recently published hypertrophic cocktail “TID” (100 nM triiodothyronine hormone, 100 ng/mL IGF1, 1 *μ*M dexamethasone) [37] for five days. HESC-cardiomyocytes in every group show distinct striations with alpha-actinin labeling (Figure 7). Further, biochemical treatment for 5 days increased cell area by 2- to 2.6-fold of untreated control with a significant increase in NRG1, IGF1 + NRG1, and TID groups (*P* < 0.01, Figure 7). These results in 2D-plated hESC-cardiomyocytes do not predict the response of the same cell type in 3D ECTs where there was no significant difference in alpha-actinin content per cardiomyocyte with growth factor stimulation (data not shown). This demonstrates the necessity of studying cardiomyocytes in the more physiological, 3D platform.

## 4. Discussion

This study describes the response of hESC-cardiomyocytes in engineered cardiac tissues (ECTs) to stimulation by developmentally important growth factors insulin-like growth factor 1 (IGF1) and neuregulin-1*β* (NRG1). The novel findings include (1) description of the amplitude and kinetics of twitch force development and the force-frequency relationship in ECTs stimulated with IGF1 and NRG1, (2) quantification of hESC-cardiomyocytes' proliferative response in 3D to IGF1 and NRG1, (3) characterization of the metabolic activity of biochemically stimulated hESC-cardiomyocytes, and (4) the observation that responses from cells in 2D and at the gene expression level do not predict 3D engineered tissue functional behavior. This study demonstrates that this ECT platform is an accessible and valuable 3D *in vitro* tool to analyze the response of hESC-cardiomyocytes to biologically important growth factors.

In an effort to develop a contractile tissue therapy for the heart, it is necessary to thoroughly understand the contractile function of engineered cardiac tissues. We used a variety of parameters to evaluate how growth factor treatment modulated ECT function. Across all groups, we observed a positive relationship between stretch and both *σ*_a_ and *v*_up_. The average active force produced by the control group (normalized only by cross-sectional area (CSA)) of 0.59 ± 0.14 mN/mm^2^ was higher than previously reported for hESC-cardiomyocyte-only, strip-like tissues [38, 39]; was equal to recently reported values [22]; and was lower than with patterned molds where overall tissue force production was normalized by CSA, porosity, and cellular orientation [40]. Notably, our tissues were formed without Matrigel or Geltrex and were cultured in serum-free conditions in contrast to these other studies, suggesting that it is possible to eliminate ill-defined protein components in the process of making engineered cardiac tissue. In our tissues, contraction kinetics are within 25% of what has been reported in both hESC-cardiomyocytes [28] and adult cardiomyocytes [41, 42]. In order to boost both passive and active mechanical properties in our ECTs to physiologically relevant levels, it will be important to create a more stiff matrix environment that more closely mimics human myocardium, and these efforts are underway by our group and others [43, 44].

Cardiomyocyte proliferation can be significantly enhanced in ECTs treated with IGF1 to 12.3 ± 1.0% (3-fold above untreated control) as evidenced histologically and metabolically, far above rates in mature, adult myocardium which have a proliferation rate of <0.1% beyond 20 years of age [45]. This phenomenon has been reported in human ESC-derived cardiomyocytes in both 2D [3] and *in vivo* [46], but had yet to be confirmed in engineered tissues *in vitro* prior to this study. A balance between proliferation and differentiation of cardiomyocytes exists in the embryonic cardiovascular system [47] where the increase in one process precludes the other. This could explain why increased cardiomyocyte proliferation rates in ECTs stimulated by IGF1 was accompanied with a significant decrease in force production, which requires assembly and maturation of the contractile lattice. Although IGF1 results in decreased twitch force amplitude in ECTs, the ability of engineered cardiac tissue to respond to proliferative stimulation is a valuable asset because of the precious nature of hESC-cardiomyocytes. Producing hESC-cardiomyocytes is an expensive endeavor, and tools to maximize yield will be key to advancing cardiac tissue engineering technologies towards therapeutic applications. Because this study focused on the detailed evaluation of ECT response to biochemical stimulation, only one treatment condition for each stimulant group was used. In the future, optimization of dose and timing could enable a more careful modulation of ECT response to growth factor stimulation to elicit a particular response. For example, IGF1 administered early in engineered tissue formation would facilitate proliferation of hESC-cardiomyocytes and could be followed by subsequent treatments or growth periods. Additionally, the B27 supplement used in this study contained insulin, which can bind and activate IGF receptors, and future studies may demonstrate increased sensitivity to IGF1 treatment with insulin-free supplement.

NRG1 treatment of ECTs produced additional novel benefits, enhancing electromechanical maturity, reflected in a nonnegative force-frequency relationship up to 3 Hz and a maximum capture rate of 2.8 Hz, and promoting metabolic activity with an increase in both mitochondrial content and oxidative phosphorylation by 1.4- and 1.5-fold over control, respectively. Metabolic sensitivity to growth factor treatment has previously been reported in hiPSC-cardiomyocytes where triiodo-L-thryonine (T3) treatment increased oxygen consumption rate without an increase in mitochondrial content but with a corresponding increase in force production [48]. Similar results were reported with a cocktail of T3, IGF1, and dexamethasone (TID) [37]. Our results also show that hESC-cardiomyocyte metabolism can be manipulated with biochemical stimulation, but in a manner that increases mitochondrial content (biogenesis). Recent work has demonstrated that mitochondria number increases during maturation of hPSC-cardiomyocytes in long-term culture, and our results suggest a means to expedite this process [49]. Currently, the field is limited to metabolic measurements of hESC-cardiomyocytes in 2D cultures, and yet we believe these data accurately represent the metabolic behavior of engineered 3D tissues because they provide plausible explanations for the phenomena observed in 3D, namely an increased rate of medium acidification (apparent with overnight color change to yellow in phenol red-containing culture medium) and decreased force production. With NRG1 stimulation, the increase in mitochondrial content and decrease in force production we observed suggests that mitochondrial maturation was achieved with NRG1 while contractile maturation was paused or hindered. This result contrasts those of T3 stimulation [48] and TID stimulation [37], suggesting that hESC-cardiomyocyte maturation is dependent upon the specific developmental stimulant. We hypothesize that metabolic maturation is required for increased contractile function of hESC-cardiomyocytes, but that removal of NRG1 stimulation to curb mitochondrial biogenesis may be required to promote contractile strength.

NRG1 stimulation in ECTs dramatically increased ANP and BNP expression greater than 50-fold over control, which suggests hypertrophic growth and is in stark contrast to the reduced maximal force we obtained with NRG1 stimulation. Spikes in ANP and BNP transcript levels are observed during important milestones in embryonic cardiac development [50]. The high levels of gene expression observed in ANP and BNP may be due to the biochemically induced developmental environment we create *in vitro* through the addition of NRG1. NRG1 has also been implicated in maintaining metabolic homeostasis [51], which could indicate that the ANP and BNP upregulation correlates with the postnatal period. Alternatively, increased ANP and BNP serum levels have been clinically associated with heart failure [52], so an alternative explanation for increased ANP and BNP levels, accompanied by decreased contractility, could be the induction of a pseudo disease state. Because NRG1 signaling is present in both development and disease [51], we hypothesize that the spikes in ANP and BNP are likely akin to normal development (e.g., ANP/BNP spike at birth due to rapid changes in hemodynamics and atrial pressure) and cardiac homeostasis because of the immaturity of these hESC-cardiomyocytes as well as the positive metabolic effects NRG1 stimulation had on ECTs. Assessing the mechanistic importance of ANP and BNP in hiPSC-cardiomyocyte maturation will require verification of protein levels and activated signaling pathways.

Our data on cardiomyocyte area versus tissue force production support the idea that the morphological response of hESC-cardiomyocytes plated on stiff surfaces in 2D is not necessarily predictive of hESC-cardiomyocyte functional maturity [53]. Biochemically treated cells showed a significantly larger area versus control in 2D, and although gene expression profiles in ECTs suggested maturity, no significant change in *α*-actinin content was observed at the tissue level as assessed by *α*-actinin staining intensity normalized per nucleus in ECTs (data not shown). Further, functional analysis of active contractile strength in ECTs showed decreased twitch force amplitude with biochemical stimulation by IGF1, NRG1, or IGF1 + NRG1. The discrepancy we find in cellular growth in 2D (increased) and force generation in 3D engineered tissue (decreased) is likely due to the different microenvironments (e.g., stiffness) that are provided to the cells in these two culture systems, which is a phenomenon that has been previously described and appears to affect cell phenotype across many cell types ranging from fibroblasts [54] and neurons [55] to primary cardiomyocytes [56]. Additionally, recent work from our group demonstrates that single hPSC-cardiomyocyte volume does not increase with area, which suggests that 2D area increase with biochemical stimulation does not truly indicate physiological hypertrophy of the cells but rather a morphological response to the microenvironment [57].

In summary, this study describes how developmentally important growth factors can be used to elicit functional responses in 3D engineered cardiac tissues (ECTs) for therapeutic applications including increased cardiomyocyte proliferation by IGF1 and metabolic maturation by NRG1. We developed and characterized an hESC-derived cardiac tissue engineering platform that incorporates static stress and electrical stimulation during culture and utilize it as a test bed for cardiac tissue maturation. ECTs responded to NRG1 and IGF1 treatment at the biomolecular, metabolic, and functional levels, demonstrating the therapeutic potential of these biochemical stimulants. We speculate that the timing and combination of developmentally pertinent growth factors including IGF1 and NRG1 will reveal methods for maximizing ECT maturity and contractile performance.

## 5. Conclusion

Our results show that growth factors identified from development, IGF1 and NRG1, modulate hESC-derived cardiomyocyte physiology in 3D engineered cardiac tissues by increasing proliferation and maturing the metabolic phenotype, respectively. IGF1 and NRG1 together synergistically acted to create a positive force-frequency response. Further, we showed that when IGF1 and NRG1 were administered to single hESC-cardiomyocytes in 2D culture, NRG1 promoted increased cell area (often referred to as hypertrophy but potentially not the case with hPSC-cardiomyocytes) [57], which was different from the 3D hESC-cardiomyocyte response as measured by force production, highlighting the necessity of 3D platforms to characterize and develop tissues for therapeutic applications.

## Figures and Tables

**Table 1:** Contractile and mechanical characteristics of engineered tissues.

Group	Fold increase	Stiffness (kPa)	*σ*_a,max_ (mN/mm^2^)	MCR (Hz)
Control	3.1 ± 0.3 (9)^†^	0.685 ± 0.265 (5)	0.59 ± 0.14 (10)	2.2 ± 0.2 (9)
IGF1	3.7 ± 0.5 (10)^†^	0.600 ± 0.177 (5)	0.22 ± 0.06 (10)^∗^	1.9 ± 0.4 (8)
NRG1	2.3 ± 0.2 (10)^†^	0.964 ± 0.158 (6)	0.21 ± 0.01 (10)^∗^	2.8 ± 0.3 (10)^∗^
IGF1 + NRG1	1.9 ± 0.2 (10)^†^	0.644 ± 0.163 (5)	0.19 ± 0.04 (10)^∗^	2.4 ± 0.27 (10)

Peak active stress (*σ*_a,max_) and maximum capture rate (MCR) were measured at 30% stretch. ^†^*P* < 0.05 versus a value of 1 (no change). ^∗^*P* < 0.05 versus control. Data are displayed as mean ± SEM with sample size displayed in parentheses.

**Table 2:** Contraction and relaxation kinetics of engineered tissues.

Group	Stretch (*n*)	*v*_up_ (mN/mm^2^/s)	*R*_50_ (ms)	*R*_90_ (ms)
Control	30% (13)	9.41 ± 2.14	167 ± 13	326 ± 24
Control	10% (12)	2.89 ± 0.93^∗∗^	113 ± 13^∗∗^	232 ± 15^∗∗^
IGF1	30% (9)	5.01 ± 1.14	144 ± 10	277 ± 24
NRG1	30% (11)	2.38 ± 0.26^∗∗^	120 ± 9^∗∗^	243 ± 25^∗^
IGF1 + NRG1	30% (13)	2.19 ± 0.45^∗∗^	139 ± 9	278 ± 28

Peak upstroke velocity (*v*_up_) and time to 50% and 90% relaxation (*R*_50_ and *R*_90_) of peak active stress, *σ*_a,max_, was measured at the indicated stretch. Sample size is displayed in parentheses. ^∗^*P* < 0.05 and ^∗∗^*P* < 0.01 versus control.
